# Sensors Based on Auxetic Materials and Structures: A Review

**DOI:** 10.3390/ma16093603

**Published:** 2023-05-08

**Authors:** Shanshan Dong, Hong Hu

**Affiliations:** School of Fashion and Textiles, The Hong Kong Polytechnic University, Hung Hom, Kowloon, Hong Kong 999077, China; shanshan.dong@connect.polyu.hk

**Keywords:** auxetic structure, resistive sensor, piezoresistive sensor, piezoelectric sensor, capacitive sensor, triboelectric sensor

## Abstract

Auxetic materials exhibit a negative Poisson’s ratio under tension or compression, and such counter-intuitive behavior leads to enhanced mechanical properties such as shear resistance, impact resistance, and shape adaptability. Auxetic materials with these excellent properties show great potential applications in personal protection, medical health, sensing equipment, and other fields. However, there are still many limitations in them, from laboratory research to real applications. There have been many reported studies applying auxetic materials or structures to the development of sensing devices in anticipation of improving sensitivity. This review mainly focuses on the use of auxetic materials or auxetic structures in sensors, providing a broad review of auxetic-based sensing devices. The material selection, structure design, preparation method, sensing mechanism, and sensing performance are introduced. In addition, we explore the relationship between the auxetic mechanism and the sensing performance and summarize how the auxetic behavior enhances the sensitivity. Furthermore, potential applications of sensors based on the auxetic mechanism are discussed, and the remaining challenges and future research directions are suggested. This review may help to promote further research and application of auxetic sensing devices.

## 1. Introduction

**Figure 1:**
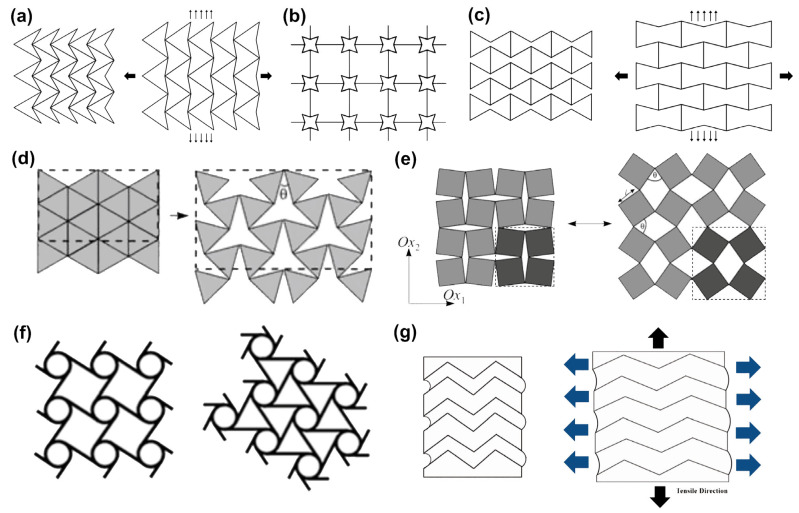
Auxetic structures: (**a**) re-entrant triangular structure, (**b**) re-entrant star structure, (**c**) re-entrant hexagonal structure, (**d**) rotating triangle, (**e**) rotating square, and (**f**) chiral structure [10]. Copyright 2010, Academic Journals. (**g**) foldable structure [11]. Copyright 2019, SAGE Publications Ltd.

Auxetic materials are unusual materials that have a negative Poisson’s ratio (NPR). When an auxetic material is stretched by an external force, the material perpendicular to the force direction expands rather than shrinks. Auxetic materials exhibit many properties superior to ordinary materials, such as high shear modulus [1,2], high impact resistance [3,4], and variable porosity [5,6], showing great application value in the fields of military, medical, and daily protection [7,8,9]. With special mechanical properties, auxetic materials have attracted increasing attention. As shown in Figure 1, auxetic structures that have been found and developed until now mainly include re-entrant structures, rotating structures, chiral structures [10], foldable structures [11], etc. The auxetic behavior of re-entrant structures such as re-entrant triangular structure (Figure 1a), re-entrant star structure (Figure 1b), and re-entrant hexagonal structure (Figure 1c) is derived from the straightening (expansion) of concave ribs. Apparently, when the entire structure is subjected to lateral or longitudinal tension, the straightening of the concave ribs causes it to expand in the other direction. Rigid triangles (Figure 1d) or squares (Figure 1e) in a rotating structure are only connected at selected vertices. When the structure is stretched in the plane, the triangles or squares rotate around these connection points, causing the structure to be auxetic. In chiral structures (Figure 1f), the straight ribs of the chiral units are connected to a central node of a circle or other geometric shapes, and the auxetic effect is achieved by releasing the ribs around the nodes under external force [10]. In contrast, the auxetic behavior of the folded structure (Figure 1g) arises simply from the expansion of the folded parts. A variety of artificial auxetic materials have been developed based on these structures, including auxetic polymers [12,13,14], auxetic composites [15,16], auxetic textiles [17,18,19,20], etc. Among these materials, a special type of auxetic material needs to be mentioned, which is 1D auxetic yarn [21,22,23]. The NPR effect of auxetic yarns is caused by an efficient structural collocation of linear materials with different Young’s modulus and stiffness rather than adopting the above-mentioned auxetic geometries. Two studies adopted re-entrant hexagonal frames for developing a resistive sensor [24] and a capacitive sensor [25], respectively. Compared with the same type of non-auxetic sensors, their sensitivities are increased by about 835 times and 3.2 times, respectively. These studies have shown that the specific deformation behavior of auxetic materials makes them sensitive to small forces or deformations, reflecting their potential in the development of sensors.

Sensors are detection devices that can sense the measured information, including strain, force, heat, light, and magnetism, and convert them into displayable electrical signals or other forms of output for information transmission, display, and storage, which are widely used in various fields involving monitoring and control. The rapid development of smart wearable electronics in recent years has drawn much attention to flexible strain sensors. Depending on the type of signal converted, the existing force/strain sensors are mainly divided into resistive [26,27,28], piezoresistive [29,30,31], piezoelectric [32,33,34], capacitive [35,36,37], triboelectric [38,39,40], and electromagnetic sensors [41,42,43]. This review focuses on the recent advances in auxetic materials or structures in sensors, especially wearable sensors. The structures, manufacturing methods, and applications of various sensors are summarized, expecting to provide some insight into the future development of auxetic sensors.

## 2. Evaluation Indicators of Auxetic Sensors

Although a standard for evaluating the performance of sensors is unavailable, indicators such as gauge factor (GF), response time, and repeatability are often used to characterize their working performance. The sensitivity of a sensor is represented by GF, which refers to the ratio of the relative change in the output signal to the corresponding strain or stress. For example, for variable resistive sensors (such as resistive and piezoresistive types), GF = (∆R/R_0_)/ε (strain sensor) [24] or GF = (∆R/R_0_)**/**∆P (pressure sensor) [44], in which ΔR/R_0_ refers to the relative resistance change, ε refers to the strain, and ∆P refers to the stress range. Other sensors have the same definition of sensitivity, but the output signal is changed to other types, such as capacitance and voltage. Response time reflects how quickly a sensor responds to strain or stress. Additionally, repeatability refers to the reproducibility of stable sensing performance of a sensor in multiple stretch-recovery or compression-recovery cycles, which indicates the durability of the sensor. In addition to sensing performance, the mechanical properties (such as breaking strength, elongation, and shear modulus) and comfort (such as flexibility, air permeability, and tactile comfort) of sensors are also important evaluation indicators that need to be considered.

## 3. Resistive Auxetic Sensos

**Figure 2:**
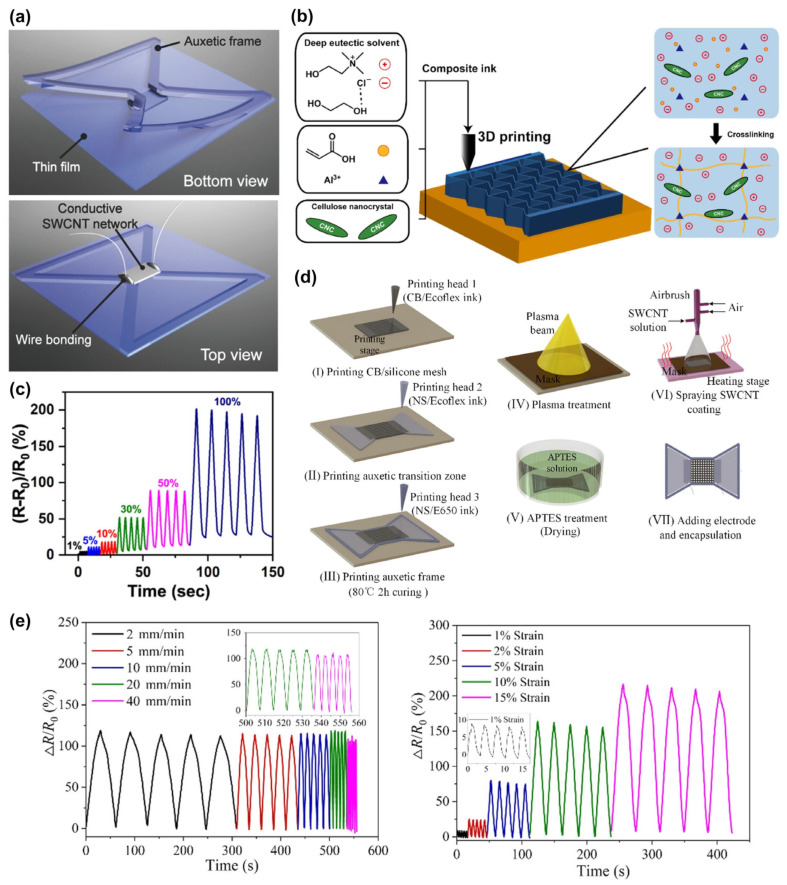
Resistive auxetic sensors: (**a**) A high-sensitivity stretchable strain sensor with a re-entrant hexagonal auxetic frame [24]. Copyright 2018, John Wiley and Sons. (**b**) A highly stretchable nanocomposite ion gel for 3D printed sensors with a re-entrant honeycomb structure and (**c**) its resistance response to small strains of 1–100% [49]. Copyright 2020, American Chemical Society. (**d**) Schematic illustration of the fabrication process of an ABSS and (**e**) its dynamic response results under stretching [50]. Copyright 2021, American Chemical Society.

Resistive sensors reflect the change in stress or strain through the change in resistance of conductive materials. When a conductor (sensing element) is subjected to an external force, its resistance changes due to changes in length, cross-sectional area, or interface with other materials, thus reflecting the physical variables. They are widely used in various fields due to their simple working principle and structures. The introduction of auxetic structures can effectively improve the sensitivity of resistive strain sensors. For example, a high-sensitivity stretchable strain sensor is shown in Figure 2a. The sensor consisted of a re-entrant hexagonal auxetic frame, a thin film, and a conductive single-walled carbon nanotube (SWCNT) network. Due to the synergistic effect of reduced structural Poisson’s ratio (PR) and strain concentration, the sensor exhibited ~24 times higher sensitivity than conventional sensors, showing great potential in medical and health monitoring [24]. An auxetic hygroscopic electrode with a serpentine network structure showed a good dynamic response and excellent air permeability, which could match the deformation of the ankle skin for electrocardiogram sensors and haptic devices [45].

Three-dimensional printing technology is often employed to fabricate resistive auxetic sensors, and a common strategy is to coat conductive materials such as graphene and carbon nanotubes (CNTs) on the 3D-printed auxetic structures. In 2019, Wong et al., developed a conductive ion gel and prepared an auxetic sensor through 3D printing technology. The auxetic structure increased the elongation by 310% compared with the traditional film. In addition, the sensor was lightweight, soft, comfortable to wear, and resistant to bending, twisting, and stretching deformations, making it easy to be integrated into wearable products [46]. Choi et al., prepared NPR structures with chiral truss and re-entrant structure auxetic lattices by 3D printing and coated the surface with castor oil-based waterborne polyurethane/graphene solution to make strain sensors. The chiral truss and re-entrant structures had a sensing range of 15% and 25%, respectively, providing a solution by adopting the thermoplastic polyurethane (TPU)-based auxetic lattice structure for various electronic sensing products made through the dip-coating method [47]. Subsequently, they proposed that the sensor fabricated by this process had superior compressive strength compared with the positive PR structure, implying its real application value [48]. Figure 2b shows a highly stretchable nanocomposite ion gel composed of cellulose nanocrystals and deep eutectic solvent for 3D printed sensors. An auxetic sensor fabricated with this type of gel and a re-entrant honeycomb structure showed a sensitive relative resistance response to small strains of 1–100% (Figure 2c) and still remained stable after 100 cycles [49]. In 2021, Wang et al., developed a novel auxetic bilayer conductive mesh strain sensor (ABSS) by 3D printing and inkjet technologies. The re-entrant structure auxetic substrate was formed by multi-material silicone rubber, and then conductive SWCNT was coated on the auxetic network as the main sensing material (Figure 2d) [50]. The dynamic response results of the sensor under stretching are shown in Figure 2e. It can be seen that the resistance change response could remain stable under different stretching speeds and strains. The high-sensitivity ABSS can monitor not only biological signals such as swallowing and respiration through muscle movements but also monitor large joint movements, which has promising application potential in sports health, medical care, etc. Li et al., adopted hybrid additive technology to manufacture an auxetic flexible strain sensor. They first used the fused deposition modeling 3D printing technology to prepare the auxetic structure made of flexible TPU and then employed the ultrasonic cavitation treatment to embed CNTs into the surface of the auxetic structure to form a conductive network. The sensor had a strain of up to 300% and a sensitivity of 2.661 [51].

**Figure 3:**
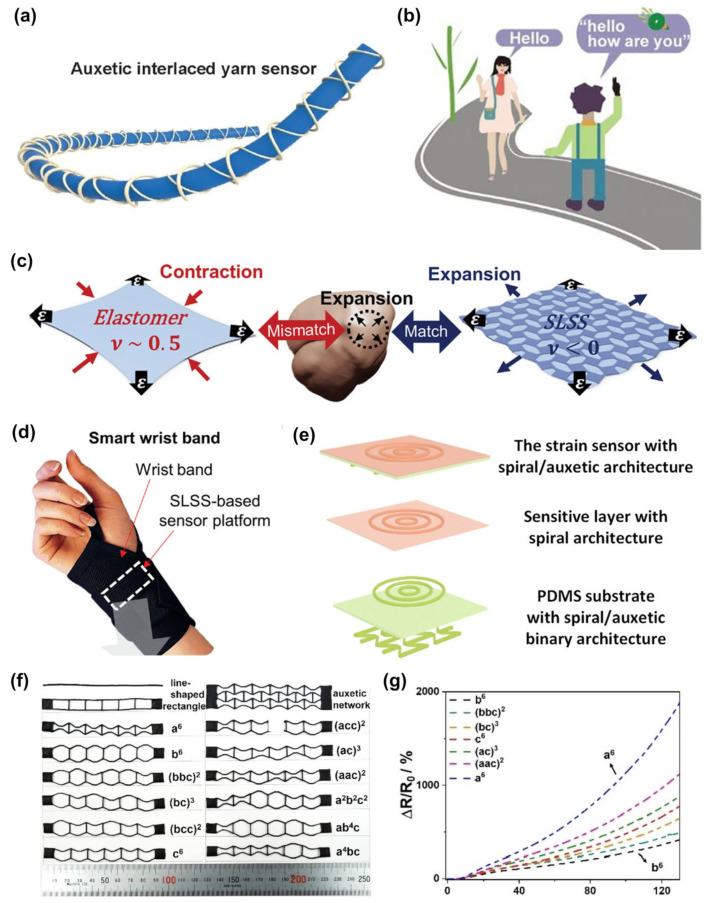
Resistive auxetic sensors: (**a**) Structural diagram of an AIYS and (**b**) its application for smart gloves with real-time conversation translation [53]. Copyright 2022, Springer Nature. (**c**) Schematic illustration of an omnidirectionally stretchable skin-like sensor and (**d**) a smart wristband system [54]. Copyright 2023, John Wiley and Sons. (**e**) Structural diagram of an ultrahigh-sensitivity strain sensor with bifacial topology [55]. Copyright 2019, American Chemical Society. (**f**) Structures of 12 different wire strain sensors and (**g**) their sensitivity performance [57]. Copyright 2022, Elsevier.

Textile-based resistive auxetic sensors have advantages in wearable applications. Ko et al., innovatively used melt electrospinning to fabricate stretchable auxetic sensors. This fiber-based sensor is lightweight, soft, and skin-friendly and can be used for hand rehabilitation exercises [52]. Figure 3a shows an all-fiber auxetic interwoven yarn sensor (AIYS) prepared by the wrapping technique, which was formed by interlocking two conductive polyamide wrapping yarns with a polyurethane (PU) core yarn at high speed. AIYS showed good sensing performance in stretching with a fast response time of 0.025 s. In addition, 16 AIYSs were embedded in the glove; after training and testing the gesture data of 26 letters (a total of 2600 data points) through deep learning, the smart sensing glove could be used for full-letter symbol recognition (99.8% recognition accuracy). All the letters could be translated from hand gestures into readable text by using the trained smart glove to achieve real-time sign language translation (Figure 3b) [53]. Kim et al., applied an auxetic reinforcement with glass fibers to an elastomer film to prepare an omnidirectionally stretchable skin-like sensor with an NPR effect (Figure 3c). Due to the fusion of the flexible substrate and auxetic reinforced structure, the sensor exhibited high stretch repeatability and good durability. In addition, the strain localization property made it suitable for various wearable sensing systems, such as a smart wristband system that monitors wrist pressure and strain in real time (Figure 3d). This omnidirectionally stretchable and highly adaptable sensing material for skin deformation showed great potential application in fields such as healthcare, virtual reality, and human–computer interaction [54].

In addition, some special structural designs can also provide effective strategies for the development of resistive sensors. For example, a bifacial topology (helical, mesh, or sinusoidal on the front and auxetic on the back) was used for ultrahigh-sensitivity strain sensors, as shown in Figure 3e. The sensor had ultrahigh sensitivity, good durability and stability, and shock resistance up to 750 N, demonstrating potential applications in monitoring weak signals from humans [55]. Mao et al., encapsulated the auxetic metamaterial in a microfluidic strain sensor designed with a core–shell seal and adjusted the deformation and strain of the microfluidic channel through the mechanical properties of the auxetic material, thereby realizing the adjustment of directional strain sensitivity [56]. In 2022, Wu et al., proposed a novel auxetic mechanical metamaterial consisting of composite wires of TPU/multi-walled CNTs (MWCNTs)/silver nanowires. Three basic building blocks (structures a, b, and c) were employed to fabricate twelve different wire strain sensors with six building blocks (Figure 3f). The influence of the proportion and arrangement of different structural units on the performance of the sensor was studied, and the results are shown in Figure 3g [57]. The sensitivity of the line strain sensor could be adjusted through the ratio and arrangement of the three structural units so that the appropriate sensor could be flexibly selected according to the actual situation. This controllable and highly sensitive sensor provided a feasible solution for high-precision sensing in various fields.

## 4. Piezoresistive Auxetic Sensor

The piezoresistive sensor also reflects the magnitude of the external force through the change in the resistance value, but its sensing components are pressure-sensitive materials, which are different from the resistive sensor. Piezoresistive materials include metals [58,59], semiconductors [60,61], graphene [62], new semiconductors [63], conductive polymer composites [64,65,66], etc. The pressure-sensitive material has little change in its own geometry under external force; instead, the resistivity changes due to the change in internal lattice parameters. Therefore, piezoresistive sensors are usually used to detect small forces or strains since their sensitivity is higher than that of resistive sensors. The introduction of auxetic materials or structures further improves the GF of piezoresistive sensors.

**Figure 4:**
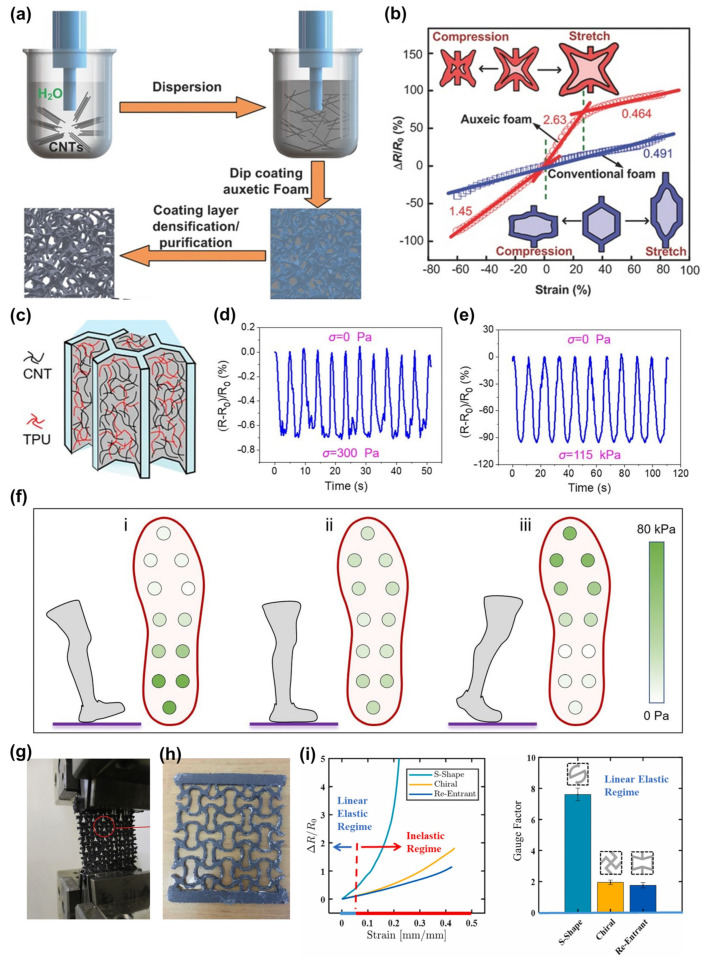
Piezoresistive auxetic sensors: (**a**) Schematic of the process for dip-coating auxetic foam with CNTs and (**b**) the sensitivity performance of the sensor [67]. Copyright 2016, John Wiley and Sons. (**c**) Schematic diagram of the pressure sensor structure based on NPR porous foam and its performance at (**d**) low and (**e**) high pressures. (**f**) Distributed sensing array composed of multiple sensors for gait monitoring when (i) heel, (ii) sole of the foot, and (iii) toe touched the ground, respectively [44]. Copyright 2023, Elsevier. (**g**) Picture of a 3D printed strain sensor made by mixing piezoresistive composite material with silicone rubber and chopped carbon fiber [69]. Copyright 2020, Elsevier. (**h**) A linear auxetic sensor with 2D constant PR [71]. Copyright 2022, Elsevier. (**i**) Performance comparison of three piezoresistive sensors with different auxetic shapes [72]. Copyright 2022, Elsevier.

Auxetic foams are often used in piezoresistive sensors. For instance, Li et al., proposed that Poisson’s ratio (PR) would affect the sensing performance of piezoresistive sensors and developed an auxetic foam sensor (AFS) with adjustable PR. Auxetic foams with different PRs were prepared first, and then CNTs were used to dip-coat conventional and prepared auxetic foams to form piezoresistive effects (Figure 4a). The sensitivity of the sensor increased significantly as the PR decreased. Compared to a conventional foam with a PR of 0.4, the AFS with a PR of −0.5 had a compressive GF of 1.45 (almost three times higher than that of conventional foams). Additionally, the tensile GF in the 30% strain range reached 2.63, five times higher than the traditional one (Figure 4b) [67]. The researchers proposed that the tunneling effect caused by the unique deformation of the auxetic foam might enhance the strain sensitivity. An AFS dip-coated by silver nanowire suspension showed good stability in both air and water and could be used in human motion detection, air pressure detection, underwater sensing, and other fields [68]. In 2023, Liu et al., employed CNT and elastic TPU as raw materials to fabricate NPR porous foams using the double-sided convergent directional freeze-casting technique. Additionally, a pressure sensor (Figure 4c) was developed based on this foam, exhibiting stable resistance change signals under both low pressure (Figure 4d) and high pressure (Figure 4e). A distributed sensing array formed by multiple sensors can be used for walking pressure and gait monitoring (Figure 4f) for exercise guidance and medical rehabilitation [44]. The manufacturing technology of this sensing element had universal adaptability and could be used to design and develop sensing systems for different occasions and needs. In addition, sensors can be prepared by 3D printing technology, such as printing the mixture of piezoresistive composite materials and silicone rubber and chopped carbon fibers into an auxetic strain sensor. As shown in Figure 4g, the sensor had an auxetic re-entrant honeycomb structure with NPR in the 6% strain range [69]. The device had high sensitivity at low strain values and could be applied to small motion monitoring or vibration detection.

For the effect of auxetic structure on the performance of piezoresistive sensors, a sensor consisting of two layers of dog-bone-shaped silicone rubber substrate and graphite powder interlayer was prepared to study anisotropic sensing behavior, and an auxetic sensing structure with constant PR was developed based on it [70]. Subsequently, Taherkhani et al., studied the importance of the PR for piezoresistive sensors and its effect on sensing characteristics and developed a linear auxetic sensor with 2D constant PR, as shown in Figure 4h. Compared with the previous conventional sensor and auxetic sensor, the GF of the developed sensor was increased by 18% and 120%, respectively, and the anisotropy of sensing performance was effectively eliminated [71]. In 2022, MWCNT/high-density polyethylene nanocomposites were developed, and 2D auxetic structures of different shapes were prepared through fused filament fabrication technology based on these materials. Among them, the S-shaped honeycomb structure exhibited higher sensitivity to strain (Figure 4i) and had excellent piezoresistive properties, with a GF of 7.6 at 4 wt% MWCNT. As the MWCNT content increased from 4% to 6%, the GF of all the auxetic structures decreased significantly, which illustrated the strong influence of the relative density of the structures on the sensing performance [72]. 

## 5. Piezoelectric Auxetic Sensor

**Figure 5:**
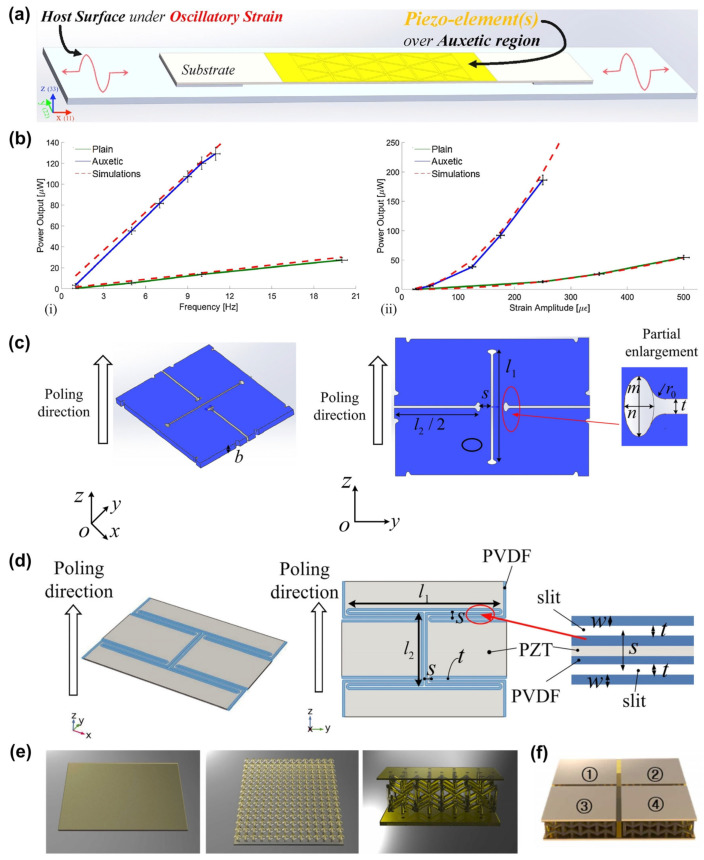
Piezoelectric auxetic sensors: (**a**) Schematic illustration of an APEH incorporating an auxetic substrate and (**b**) power output of optimized APEH at different (i) frequencies and (ii) strain amplitudes [81]. Copyright 2018, Elsevier. (**c**) Schematic diagram of the piezoelectric model based on ultra-low porosity auxetic piezoelectric ceramics [82]. Copyright 2020, John Wiley and Sons. (**d**) Schematic illustration of piezoelectric auxetic composites formed by introducing hollow PVDF into PZT matrix [83]. Copyright 2021, John Wiley and Sons. (**e**) Three-dimensional models of different structures based on piezoceramic polymer composites and (**f**) a tactile position tracking sensor realized based on the auxetic model with four square patterns [85]. Copyright 2023, Elsevier.

Piezoelectric materials are crystalline materials with a piezoelectric effect, usually including piezoelectric crystals (single crystals) [73,74], piezoelectric ceramics (polycrystalline semiconductors) [75,76,77], and polymer piezoelectric materials [78,79]. Under the external force, the piezoelectric material generates a polarization phenomenon inside to be charged and generate a voltage signal. Piezoelectric ceramics are popular due to their unique electromechanical coupling properties. However, the practical application of monolithic piezoceramics is limited due to its high impedance and brittleness. Compared with ordinary piezoelectric ceramics, the piezoelectric auxetic lattice can significantly amplify the triggered effective strain (30–70 times) and the piezoelectric coupling coefficient of the lattice, which is helpful for the preparation of more sensitive piezoelectric strain sensors [80]. Ferguson et al., developed an auxetic piezoelectric energy harvester (APEH) by introducing an auxetic substrate (Figure 5a). Through finite element modeling analysis, APEH showed 11.5 times power higher than traditional PEH. Moreover, the tolerance to stress was also improved. The actual power output of the optimized APEH also matched the predicted results (Figure 5b) [81]. In 2020, Tang et al., showed for the first time the figure of merit of an auxetic piezoceramic with ultra-low porosity. As shown in Figure 5c, the auxetic piezoelectric model had a rotated square structure, and the effects of PR, elastic coefficient, piezoelectric stress coefficient, dielectric coefficient, and quality factor on auxetic piezoelectric ceramics were studied [82]. Subsequently, the effect of PR on the properties of piezoelectric composites was further investigated. As shown in Figure 5d, the composite was formed by introducing hollow polyvinylidene difluoride (PVDF) into the lead zirconate titanate (PZT) matrix. Through equation calculation and finite element analysis, some main conclusions were obtained: (1) The piezoelectric stress and permittivity in transversely polarized materials were generally more sensitive to geometrical and compositional parameters than in longitudinally polarized piezoelectric composites. (2) The auxetic structure could improve the ductility of existing piezoelectric composites. (3) The electromechanical properties of this piezoelectric composite material were more customizable than traditional porous piezoelectric materials. These findings provided some effective guidance for the design and development of new auxetic piezoelectric metamaterials [83]. A piezoelectric energy harvester combining the two key topologies of auxetic and kirigami was also developed. Through the analysis and verification of the simulation model, it was proved that its output efficiency had been significantly improved compared with the traditional structure [84]. In 2023, Chang and his team designed 3D-printable piezoceramic-polymer composites that exhibited good interfacial adhesion, high dispersion stability, low viscosity, and smooth surfaces. The 3D models of different structures designed based on this material simulation are shown in Figure 5e. Through COMSOL simulation, it was concluded that the piezoelectric performance of the auxetic structure would be effectively improved. Finally, a tactile position tracking sensor, which exhibited good flexibility and stretchability [85], was realized based on the auxetic structure (Figure 5f). Three-dimensional-printed piezoelectric composites with auxetic structures have shown great potential in areas such as wearable sensing, tactile sensing, and pressure monitoring. 

## 6. Capacitive Auxetic Sensor

**Figure 6:**
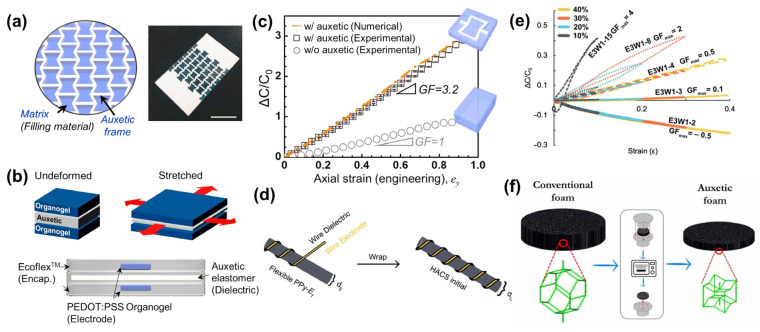
Capacitive auxetic sensors: (**a**) Schematic illustration of a concave hexagon-based NPR mechanically programmable continuous elastomer and (**b**) a stretchable strain sensor based on this auxetic elastomer. (**c**) Capacitance change in the auxetic sensor under tension [25]. Copyright 2019, Elsevier. (**d**) Structure diagram of the HACS and (**e**) the sensitivity performance of sensors with different structural parameters [86]. Copyright 2022, John Wiley and Sons. (**f**) Capacitive strain device incorporating auxetic PU foam with 3D re-entrant cells [88]. Copyright 2023, IOP Publishing Ltd.

Capacitive sensors are devices that convert physical or mechanical quantities into capacitance changes, which are widely used in the measurement of displacement, vibration, speed, pressure, etc. The sensitivity of capacitive sensors has been limited due to their structure and working principle. Conventional materials have a positive PR, and the area expansion of the capacitor element is limited to less than the amount of applied strain, so the sensor cannot achieve a GF greater than 1 [86]. Shintake et al., integrated layered auxetic structures (rotating squares) into a capacitive sensor by laser cutting to improve its sensitivity. The GF of the auxetic sensor reached 1.61, which was higher than 0.86 for the non-auxetic structure, and both had good repeatability and durability [87]. Lee et al., designed an NPR mechanically programmable continuous elastomer based on re-entrant hexagons, which consisted of TPU as the frame and soft silicone rubber as the filling material, as shown in Figure 6a. A stretchable strain sensor was developed by linking a PEDOT:PSS organogel conductor to an auxetic elastomer (Figure 6b). Figure 6c shows the capacitance change in the auxetic sensor under stretching. It is obvious that the introduction of the auxetic elastomer dielectric greatly improved the capacitance change sensitivity [25]. This programmable mechanical hyperelastic material incorporating an auxetic framework showed great significance in the field of mechanical sensing. In 2022, Cuthbert et al., developed a helical auxetic yarn capacitive sensor (HACS) for the first time, breaking through the sensitivity limit of the capacitive sensor. As shown in Figure 6d, HACSs with different helix lengths and diameter ratios were prepared by using enameled copper wire with a diameter of 0.11 mm as the wrapping yarn and polypyrrole-polymerized polyester-wrapped elastic fiber as the core yarn. While the sensor with a helix length of 8 mm (E3W1-8) increased its GF beyond the theoretical limit of 2, the sensor with a helix length of 15 mm (E3W1-15) achieved a maximum GF of up to 4 (Figure 6e) [86]. The as-prepared HACSs were generally highly stable and flexible, making them easy to be integrated into textiles and showing great application potential in wearable sensing. Subsequently, a mathematical model was first proposed to simulate the auxetic behavior in capacitive strain sensors, and then a capacitive strain device was developed by combining the auxetic PU foam of 3D re-entrant cells (Figure 6f) with polydimethylsiloxane/CNT electrodes. The measured GF of the auxetic sensor in the 10% strain range was −2.8, which was consistent with the proposed model [88]. This study provided an effective reference for the future design and development of auxetic soft strain sensors.

## 7. Triboelectric Auxetic Sensor

**Figure 7:**
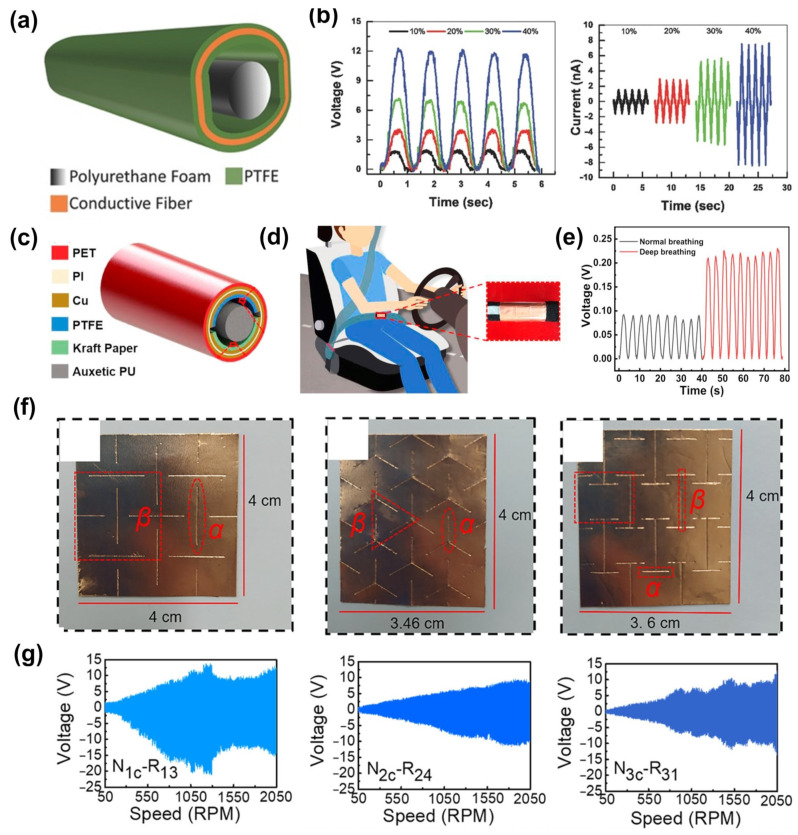
Triboelectric auxetic sensors: (**a**) Schematic of the first TENG using auxetic foam and (**b**) its open-circuit voltage and short-circuit current under different tensile strains [99]. Copyright 2017, John Wiley and Sons. (**c**) Structure of the TENG based on auxetic foam and (**d**) its application in a seat belt and (**e**) the electrical output of the driver under different breathing states [100]. Copyright 2019, Elsevier. (**f**) Auxetic TENGs with rotating quadrilateral structure, rotating triangular structure, and “I”-shaped structure. (**g**) Voltage output of sensors with different parameters under different speeds [102]. Copyright 2022, American Chemical Society.

A triboelectric nanogenerator (TENG) is a new type of green energy device that converts mechanical energy into electrical energy through triboelectrification and electrostatic induction [89]. Its mechanism as a sensor is to reflect the magnitude and form of the force through the strength and waveform of the electrical signal (voltage or current) generated. The wide range of materials and facile fabrication process makes it easy to be flexible [90,91], stretchable [92,93], breathable [94,95], and washable [96]. Moreover, some textile-based TENGs can be seamlessly integrated into clothing [97,98], showing great potential in wearable sensors. However, the sensitivity of conventional materials is often limited, and auxetic materials or structures exhibit considerable advantages in enhancing the sensitivity of TENGs. In 2017, Zhang et al., pioneered the application of auxetic materials to a contact TENG strain sensor. As shown in Figure 7a, the auxetic sensor consisted of a core-auxetic PU foam and a shell-polytetrafluoroethylene (PTFE)-conductive fabric-PTFE layer. When the tension was applied to the auxetic foam, the foam expanded and contacted the PTFE layer to triboelectrically electrify. Its open-circuit voltage and short-circuit current sensitivities to strain are shown in Figure 7b, implying a higher strain sensitivity than other TENGs [99]. In addition, the auxetic foam had high resistance to indentation and shear, which could provide better durability for the sensor. Feng et al., applied an auxetic foam-based TENG (Figure 7c) to the belly of the seat belt to monitor the driver’s breathing state (Figure 7d,e). The sensor showed a high sensitivity of 0.89 V/cm^2^ in a strain range of 40–100%, which could be used to provide an alarm in the event of danger [100]. For use in wearable products, textile-based TENG sensors have great advantages. The auxetic yarn made of TPU as the core yarn and conductive polyamide as the wrap yarn prepared by high-speed ring spinning method was inserted into a woven fabric as weft yarn to form a TENG. The TENG sensor had distinctly varying electrical outputs under different stretching strains and can be used as stretch sensors for smart sports products, such as being embedded into a self-counting yoga elastic band [101]. Due to the feasibility of mass production, this triboelectric auxetic sensing yarn offered a promising direction for self-powered sensors. In 2022, Ye et al., prepared auxetic TENGs with three different structures (rotating quadrilateral structure, rotating triangular structure, and “I”-shaped structure) (Figure 7f) to study the influence of structural parameters on sensor performance. As shown in Figure 7g, the sensor N_1c_-R_13_ with a rotating quadrilateral structure and a ratio of cutting marks to cell structure size (α/β) of 1.4/2.0 showed the largest voltage output [102].

The characteristics of the main sensors mentioned above, including working mechanisms, auxetic materials and structures used, fabrication methods, sensing performance, and applications, are summarized in Table 1. In summary, the conductive materials employed in auxetic sensors mainly include metals (Ag nanowires, Ag/Cu coatings, and liquid metals), carbon-based conductive materials (MWCNT, CNT, graphite powder, graphene, and carbon fibers), and conductive ionogel. For the manufacture of auxetic sensors, the simplest and universally applicable method is to add sensing materials on the substrate of auxetic materials (mainly made by 3D printing or laser cutting) by coating, bonding, filling, assembling, and other methods (except for the production of auxetic yarn sensors). The increase in sensitivity is evident on the resistive sensor, even reaching an improvement of about 835 times. The piezoresistive auxetic sensor achieves a working strain range (<6%) that is difficult for conventional piezoresistive sensors. Piezoelectric auxetic sensors improve the problem of insufficient extensibility and can achieve tailorable electromechanical properties, although the sensitivity improvement is less. Capacitive auxetic sensors break through the sensitivity limit of traditional capacitive sensors and realize a GF higher than 1. The sensitivity of the triboelectric auxetic sensor is not good enough; however, its fabrication simplicity and the deformation comfort provided by the auxetic substrate make it suitable for wearable systems. The potential applications of auxetic sensors are mainly concentrated in wearable fields such as motion monitoring, vital sign detection, soft robotics, and human–computer interaction, which implies greater requirements for the comfort of the sensors.

## 8. Challenges and Future Prospects

In this review, various types of sensors employing auxetic materials and structures are introduced, and their materials, fabrication methods, behavior, and possible applications are discussed in detail. The auxetic sensors mentioned in this review generally have broad application prospects in wearable products because they usually exhibit good stretchability and sensitivity. Despite quite a few positive results on auxetic sensors, there is still a large gap between research and practical applications, and several challenges are discussed below.

(1) The mechanism of how the auxetic material improves sensitivity has not been studied. Although some researchers have proposed that the special mechanical properties of auxetic materials may amplify small force or deformation and thereby enhance the corresponding electrical response signal, the specific details have not been systematically investigated and discussed.
(2) There is no systematic study on the relationship between auxetic properties and sensing performance. Several studies have proposed a relationship between NPR and sensitivity based on a specific sensor structure, but still, no generally applicable rules or relationships have been provided, which requires more samples and data.
(3) Insufficient utilization of auxetic structures. Currently, auxetic foams and re-entrant hexagonal structures are mostly used in sensors, while other auxetic structures, including rotating structures, chiral structures, and foldable structures, have little or no proportion. Researchers are expected to create more high-performance sensors based on various auxetic structures, which will not only help in the development of breakthrough sensors but also expand the library of auxetic sensors for subsequent systematic studies (including the studies on the first and second challenges).
(4) The comfort of the auxetic sensor has not been carefully considered. Auxetic sensors show great potential in wearable applications such as sports rehabilitation, vital sign detection, and human–machine interaction due to their enhanced extensibility and sensitivity. However, few studies focused on their wearing comfort, including softness, skin-friendliness, breathability, etc.

In general, auxetic sensors have effectively improved comfort and sensitivity compared with traditional sensors, showing great application prospects in many fields, especially in wearable systems. The development of the auxetic sensor is still in its infancy, and more research is expected to promote its commercial application.

## 9. Conclusions

Auxetic materials or structures can effectively improve the sensitivity of sensors due to their special mechanical properties. Currently, the most popular auxetic structure in sensors is the re-entrant structure, especially the re-entrant hexagon, because it is relatively stable and easy to be realized. The generally applicable sensor preparation method is to add sensing materials on the substrate of auxetic materials (except triboelectric auxetic sensors). In addition to the five types mentioned, the auxetic structure has also been applied to other types of sensors, such as electromagnetic sensors, but there are few related studies at present. Auxetic materials have good shape adaptability and protective properties, which can provide better comfort and protection in wearable applications. Therefore, auxetic sensors have natural advantages in smart wearable applications. However, the current research on auxetic sensors has just started, and their comfort property has not been seriously taken into consideration at present. Considering the flexibility in material selection, resistive and triboelectric sensors are more likely to be flexible, stretchable, breathable, and easily integrated into wearable products. More research on wearable auxetic sensors could be conducted in the future.

## Figures and Tables

**Table 1:** A summary of sensors based on auxetic materials and structures.

Type	Electrical Material	Auxetic Material/Structure	Fabrication Method	GF	Response Time	Application	Ref.
Resistive	SWCNT	PDMS re-entrant hexagonal frame	3D printing, self-pinning	≈835	-	Pulse detection	[24]
	Ag nanowires	PEG-DA gel serpentine network	UV crosslinking, spraying	6.17	-	ECG sensor, haptic device	[45]
	Ionogel	Re-entrant hexagonal structure	3D printing	-	-	Wearable strain sensor	[46]
	Nanocomposite ionogel	Re-entrant honeycomb	3D printing	3.3	-	Wearable motion sensor	[49]
	SWCNT	Silicone rubber re-entrant frame	3D printing, ink spraying	~ 13.4	160 ms	Monitoring of swallowing, respiration, and joint bending	[50]
	Silver-coated PA yarn	Auxetic yarn	Yarn-wrapping technology	-	25 ms	Sign-language translation glove	[53]
	Ag/CNT	Glass fiber-based elastomer film	Deposition	300	-	Smart wristband	[54]
	Polyethylenimine-reduced graphene oxide	PDMS re-entrant hexagonal film	Laser scribing, coating	~1744	-	Monitoring human motions	[55]
	Graphene	Polyimide re-entrant hexagonal film	Laser cutting, casting, injecting, bonding	~10	-	Monitoring wrist movements	[56]
	TPU/ MWCNTs/ AgNWs	2D auxetic network	Injecting, drying	21.8	-	Weight and displacement recognition	[57]
Piezoresistive	CNT	Auxetic foam	Coating	2.63	-	Smart helmet, pulse monitoring, human–machine interaction	[67]
	CNT	Auxetic porous foam	Ambilateral convergent directional freeze casting methodology	−5.4 kPa^−1^	-	Gait monitoring	[44]
	Carbon fiber	Re-entrant hexagonal polymer frame	3D printing	Sensitive at low strain (<6%)	-	Pulse and vibration detection	[69]
	Graphite powder	Silicon/graphite powder-based auxetic frame	Through a mold	18% increase	-	-	[71]
	MWCNT	S-shaped cellular structure	Fused filament fabrication, 3D printing	7.61	-	Wearable electronics and self-sensing prosthetics	[72]
Piezoelectric	Auxetic piezoelectric ceramics with ultra-low porosity		Model and geometry analysis	Highly tailorable electromechanical properties		Instruct design and application of the ultra-low porous auxetic piezoelectric materials.	[82]
	PZT/PVDF auxetic piezoelectric composite		Finite element analysis				[83]
	Ceramic piezoelectric composite	3D re-entrant hexagonal structure	3D printing	Output voltage 6 V (three times higher than flat plate)		Tactile location-tracking sensor	[85]
Capacitive	PEDOT:PSS/poly-acrylamide conductive organogel	Auxetic elastomer frame (re-entrant hexagonal)	Cutting, filling	3.2	-	Wearable devices	[25]
	Copper wire	Helically wound yarn	Wrapping	4	-	Wearable devices	[86]
	Liquid metal (eutectic gallium-indium)	Silicone elastomer rotating structure	Laser cutting, assembling	1.61	-	Soft robotic system	[87]
	CNT/PDMS/MEP conductive paste	Anisotropic auxetic PU sponge	Thermal compression, dipping	−2.8	-	Smart wearables and perceptive soft robots	[88]
Triboelectric	Conductive fiber, PTFE	Auxetic PU foam (core)	Assembling	1.6 V cm^−2^	-	Motion sensing, weight Sensing	[99]
	Cu, PTFE, kraft paper	Auxetic PU foam (core)	Assembling	0.89 V cm^−2^	~52 ms	Smart seat belt	[100]
	Silver-coated PA yarn, silicone rubber	Auxetic yarn	High-speed ring spinning, weaving	Output voltage 0.545 V	-	Self-counting yoga elastic band	[101]
	Cu, PVDF, PDMS	Rotating structures and an “I”-shaped structure	Cutting, heating	-	-	Monitoring human motions	[102]

## Data Availability

No new data were created or analyzed in this study.
